# The Prevalence of Hyperuricemia Sharply Increases from the Late Menopausal Transition Stage in Middle-Aged Women

**DOI:** 10.3390/jcm8030296

**Published:** 2019-03-02

**Authors:** Sung Kweon Cho, Cheryl A. Winkler, Soo-Jin Lee, Yoosoo Chang, Seungho Ryu

**Affiliations:** 1 Department of Health Sciences and Technology, SAIHST, Sungkyunkwan University, Seoul 06351, Korea; sungkweon.cho@nih.gov; 2 Molecular Genetic Epidemiology Section, Basic Research Laboratory, Center for Cancer Research, National Cancer Institute, Frederick, MD 21702, USA; winklerc@mail.nih.gov; 3 Department of Occupational and Environmental Medicine, College of Medicine, Hanyang University, Seoul 04763, Korea; sjlee@hanyang.ac.kr; 4 Department of Clinical Research Design & Evaluation, SAIHST, Sungkyunkwan University, Seoul 06351, Korea; 5 Center for Cohort Studies, Total Healthcare Center, Kangbuk Samsung Hospital, Sungkyunkwan University School of Medicine, Seoul 04514, Korea; 6 Department of Occupational and Environmental Medicine, Kangbuk Samsung Hospital, Sungkyunkwan University School of Medicine, Seoul 03181, Korea

**Keywords:** hyperuricemia, menopause, late menopausal stage

## Abstract

The impact of menopausal transition on change of serum uric acid level remains unknown. The present study evaluated the relationship of menopausal stages with prevalent hyperuricemia in middle-aged women. This cross-sectional study included 58,870 middle-aged Korean women, aged ≥40, who participated in a health examination from 2014 to 2016. Menopausal stages were obtained with a standardized, self-administered questionnaire and were categorized according to the criteria of the Stages of Reproductive Aging Workshop (STRAW+10). Hyperuricemia was defined as a serum uric acid level of ≥6 mg/dL. The prevalence of hyperuricemia increased as menopausal stage increased. The multivariable-adjusted odds ratios (95% confidence intervals) for prevalent hyperuricemia comparing early transition, late transition, and post-menopause to pre-menopause were 1.19 (0.80–1.77), 2.13 (1.35–3.36), and 1.65 (1.33–2.04), respectively. This association was stronger among non-obese compared to obese participants and in those with low high-sensitivity C-reactive protein (hsCRP) levels (<1.0 mg/L) compared to those with elevated hsCRP levels of ≥1.0 mg/L (*p* for interaction = 0.01). In this large sample of middle-aged women, the prevalence of hyperuricemia significantly increased from the menopausal stage of late transition, independent of potential confounders. Appropriate preventive strategies for reducing hyperuricemia and its related consequences should be initiated prior to menopause.

## 1. Introduction

Until the early 1800s, uric acid was thought to be a biologically inert waste product, but hyperuricemia is increasingly considered a potential risk factor for various chronic conditions such as chronic kidney disease, cardiovascular disease, and metabolic syndrome, and has a well-established causal role in gout [1,2,3]. Hyperuricemia is a common biochemical abnormality resulting from excessive uric acid production or impaired clearance of uric acid [4,5]. Although its pathophysiology is not fully understood, genetic, comorbid disease-related, and environmental (drug, diet, and toxic exposure-induced) factors are involved in hyperuricemia [6,7,8].

Menopause is defined as a point in time retrospectively after 12 months of amenorrhea [9]. The menopausal process includes a gradual change from pre- to post-menopause, rather than two simple states of pre- and post-menopause; thus, the 2011 Stages of Reproductive Aging Workshop (STRAW+10) updated guidelines to provide a comprehensive staging system for the process of menopause [9], in which transitional stages are classified as pre-menopause, early menopausal transition, late menopausal transition, and post-menopause based on changes in bleeding flow and intervals. During the menopausal transition, changes in estrogen and progesterone levels affect not only vasomotor symptoms, sexual dysfunction, osteoporosis, and cardiovascular disease (CVD) [10], but also uric acid level. An association between menopausal status and hyperuricemia has been previously examined [11,12]. A study using the Third National Health and Nutrition Examination Survey showed a positive and independent association of menopause with hyperuricemia and gout [11,12]. In another study, menopause was independently associated with a high level of uric acid after adjustment for age and body mass index (BMI) [13]. Obesity is related to an increased risk of menstrual disorders and decreased fertility in premenopausal women, and to increased cardiovascular risk in postmenopausal women [14]. The prevalence of obesity, which is a risk factor for hyperuricemia, increases during the menopausal period and also varies from country to country [15,16,17]. According Organisation for Economic Co-operation and Development (OECD) 2016 statistics, the prevalence of overweight (BMI >25 kg/m^2^) is variable by the countries (23.4% in Korean women, 37.0% in Mexican women (the highest), and 16.6% in Japanese women). Until now, the change in uric acid level according to menopausal transition stage has not been evaluated considering whether the association between menopausal transition stage and hyperuricemia differs by obesity. A better understanding the relationship between menopausal stage and hyperuricemia helps for identifying optimal timing for preventive measures for hyperuricemia. 

Therefore, we evaluated the association of menopausal stages based on STRAW+10 (pre-menopause, early- and late-menopausal transition, and post-menopause) with the prevalence of hyperuricemia in middle-aged Korean women.

## 2. Methods

### 2.1. Study Population

This study is a part of the Kangbuk Samsung Health Study which is a cohort study of South Korean adults who participated in a health examination annually or biennially at Kangbuk Samsung Hospital Total Healthcare Centers in Seoul and Suwon, South Korea [18,19]. The majority (≥80%) of participants were employees or their spouses from various companies or local governmental organizations [19]. In South Korea, the Industrial Safety and Health Law requires annual or biennial health screening examinations for all employees, offered free of charge [19].

The present study included 75,166 women, age of ≥40, who participated in a health examination from 2014 to 2016. A total of 16,296 subjects were excluded for the following reasons: a history of hysterectomy or bilateral oophorectomy (*n* = 5304), a history of cancer (*n* = 4541), radiation- or chemotherapy-related menopause (*n* = 906), other type of artificial menopause (*n* = 625), current use of hormone therapy (*n* = 1792), current use of oral contraceptives (*n* = 295), low estimated glomerular filtration rate (eGFR) level (<60 mL/min) (*n* = 315), and missing information on history of menopause or uric acid level (*n* = 6492). Some individuals met more than one exclusion criterion: thus, 58,870 participants was eligible in the final analysis. The Institutional Review Board of Kangbuk Samsung Hospital approved this study and waived the requirement for informed consent because of use of non-identified retrospective data routinely collected during the health checkup program (IRB No. KBSMC 2016-09-040). We confirm that all methods were performed in accordance with the relevant guidelines and regulations.

### 2.2. Data Collection

Information regarding reproductive factors (parity, regularity and interval of menstrual cycles, menarcheal age, use of oral contraceptives and use of hormone therapy), demographic characteristics, lifestyle factors, medical history, and medication use was obtained with a standardized, structured, self-administered questionnaire. Parity was defined as the number of previous pregnancies including live births and stillbirths. Menopausal stages were categorized by the criteria of the STRAW+10 as follows: (1) pre-menopause; (2) early menopausal transition was defined as a persistent difference of ≥7 days in length of consecutive cycles; (3) late menopausal transition was defined as the occurrence of amenorrhea of ≥60 days; and (4) post-menopause was defined as absence of a menstrual period for ≥12 months [9,20]. Dietary intake was evaluated using a Korean version of 106-item self-administered food frequency questionnaire (FFQ) [21]. Total energy intake was calculated on the basis of the standardized food-composition database. Physical activity level was assessed using the Korea-validated version of the International Physical Activity Questionnaire (IPAQ) short form and was categorized as being inactive, minimally active, and health-enhancing physically active (HEPA) [22].

Height, weight and blood pressure (BP) were measured by trained nurses. Obesity was defined as BMI ≥25 kg/m^2^ according to Asian-specific criteria [23]. Hypertension was defined as a systolic BP ≥140 mmHg, diastolic BP ≥90 mmHg, or current use of BP-lowering medication.

Fasting blood tests included total cholesterol, low-density lipoprotein (LDL) cholesterol, high-density lipoprotein (HDL) cholesterol, triglycerides, alanine transaminase (ALT), gamma-glutamyl transferase (GGT), creatinine, glucose, uric acid, insulin, and high sensitivity C-reactive protein (hsCRP). Serum uric acid level was measured enzymatically using an automatic analyzer (Modular DP analyzer, Roche Diagnostics, Tokyo, Japan). Homeostatic model assessment of insulin resistance (HOMA-IR) was used to estimate insulin resistance and was calculated as fasting blood insulin (mU/mL) × fasting blood glucose (mmol/L)/22.5. Estimated GFR was calculated using the Chronic Kidney Disease Epidemiology Collaboration equation [24]. Diabetes was defined as a fasting serum glucose ≥ 126 mg/dL, A1c ≥ 6.5%, or current use of insulin or anti-diabetes medications. Hyperuricemia was defined as serum uric acid level of ≥6 mg/dL since there is no universally accepted definition of hyperuricemia solely based on uric acid level due to differences related to age and sex [25].

### 2.3. Statistical Analysis

Characteristics of the study participants were presented according to menopausal stage using descriptive summary statistics. The number of categories was treated as a continuous variable in regression models in order to test for linear trends.

To assess the relationship of prevalent hyperuricemia with menopausal stages, a logistic regression model was used to estimate the odds ratios (ORs) with a 95% confidence internal (CI) for hyperuricemia in menopausal stages compared with pre-menopause. First, the model was initially adjusted for age and then model 1 was further adjusted for center, year of examination, BMI (continuous), educational level (high school graduate or less, community college or university graduate, graduate school or higher, or unknown), smoking status (never, former, current, or unknown), physical activity (inactive, minimally active, HEPA, or unknown), alcohol intake (0, <10, ≥10 g/day, or unknown), total energy intake (in quintile or missing), parity (none, 1 to 2, ≥3), and menarcheal age. Model 2 was additionally adjusted for medication for hypertension and levels of glucose, LDL-cholesterol, triglycerides, HDL-cholesterol, systolic BP, HOMA-IR, eGFR, and hsCRP.

Additionally, we performed subgroups analyses stratified by BMI (<25 vs. ≥25 kg/m^2^), ever smoking (no vs. yes), alcohol consumption (<10 vs. ≥10 g of ethanol/day), physical activity (inactive vs. minimally active vs. HEPA), HOMA-IR (<2.5 vs. ≥2.5), and hsCRP (<1.0 vs. ≥1.0 mg/L). Likelihood ratio tests were used to test interactions by subgroup comparing models with and without multiplicative interaction terms.

Lastly, for age-matched analyses, each woman with hyperuricemia was matched with a randomly selected woman without hyperuricemia, of the same age, regardless of menopausal stage. Age-matched conditional logistic analysis of hyperuricemia was conducted with adjustment for center, year of examination, education, alcohol consumption, smoking status, physical activity, total energy intake, BMI, parity, and menarcheal age.

The statistical analysis was conducted using STATA, version 15.0 (StataCorp LP, college station, Texas, TX, USA). The *p*-value for statistical significance was defined as *p* <0.05 for two-tailed test.

## 3. Results

Table 1 shows the characteristics of 58,870 women by menopausal stage. The mean (SD) ages and BMIs of the study participants were 46.9 years (7.3) and 22.5 kg/m^2^ (3.1), respectively. The proportions of women in pre-menopause, early transition, late transition, and post-menopause were 65.2%, 9.6%, 4.4%, and 20.8%, respectively, and mean (SD) uric acid levels in the corresponding menopausal stages were 4.2 (0.9), 4.2 (0.9), 4.4 (1.0), and 4.5 (1.0) mg/dL, respectively. BP, glucose, total cholesterol, LDL-cholesterol, triglycerides, ALT, GGT, HOMA-IR, hsCRP and total energy intake were positively associated with menopausal stage, while eGFR and HDL-cholesterol were inversely associated with menopausal stage (*p* for trend <0.05). However, current smokers did not show any trend by menopausal stages (*p* for trend = 0.387). 

A total of 2155 (3.7%) women with hyperuricemia were identified among the 58,870 women. The prevalence of hyperuricemia increased with stage of menopause (*p* for trend <0.001) (Table 2). After adjustment for age, center, year of examination, education, BMI, smoking status, physical activity, alcohol consumption, total energy intake, parity, and menarcheal age, the multivariable-adjusted ORs (95% CIs) for hyperuricemia comparing early transition, late transition, and post-menopause to pre-menopause were 1.18 (1.00–1.40), 1.67 (1.38–2.03), and 1.96 (1.66–2.31), respectively. To explore whether the association was mediated by metabolic parameters, insulin resistance, or inflammation, we performed additional analyses while adjusting for medications for hypertension, glucose, systolic BP, triglycerides, HDL-cholesterol, LDL-cholesterol, HOMA-IR, eGFR, and hsCRP. In this mediation analysis, the association between menopausal stage and prevalent hyperuricemia was attenuated but persisted. The corresponding ORs (95% CIs) for hyperuricemia comparing early transition, late transition, and post-menopause to pre-menopause were 1.19 (0.80–1.77), 2.13 (1.35–3.36), and 1.65 (1.33–2.04), respectively (*p* for trend <0.001) (Table 2). Age-matched conditional logistic analysis of hyperuricemia by menopausal stage also showed that the prevalence of hyperuricemia significantly increased with stage of menopause (*p* for trend <0.001) (Table 3).

The prevalence of hyperuricemia statistically increases by menopausal stages in each subgroup stratified by BMI, ever smoking, alcohol intake, physical activity, HOMA-IR and hsCRP except the smoking group (*p* for trend <0.05). The relationship of menopausal stage with hyperuricemia was similar across various subgroups, except for BMI and hsCRP. The associations between menopausal stage and hyperuricemia was stronger (Table 4) in non-obese compared to obese participants (*p* for interaction <0.001) and in those with low hsCRP level (<1.0 mg/L) compared to those with hsCRP level of ≥1.0 mg/L (*p* for interaction = 0.01).

## 4. Discussion

In this large sample of middle-aged, Korean women, menopausal stage was significantly associated with increased prevalence of hyperuricemia in a dose-dependent manner, and a significantly increased prevalence of hyperuricemia started at the late transition stage. This association was still observed after adjustment for potential confounders. This is the first study examining the prevalent hyperuricemia according to the newly updated menopausal stages (STRAW+10) [9].

A study from the Nurses’ Health Study of 92,535 women showed that postmenopausal status was associated with a higher risk of incident gout than premenopausal status [12]. Hak et al. found that menopause was independently associated with hyperuricemia using the Third National Health and Nutrition Examination Survey [11], after adjustment for covariates including age, BMI, serum creatinine, use of diuretics, diabetes, and hypertension. Using the same data, Krishnan et al. showed that aging, but not menopause, was associated with an increased prevalence of hyperuricemia [26]. All these studies did not differentiate the transitional stage of menopause due to their method of data collection. The KORA F4 study of 1530 women in Southern Germany by Stöckl et al. demonstrated that reproductive factors such as postmenopausal status, menarcheal age, and a history of oral contraceptive use were independently associated with hyperuricemia [27]. That study also could not differentiate the menopausal transition stage and was limited by a lack of dietary factors as well as the relatively small sample size.

The biological mechanism underlying the change of uric acid level is most likely the influence of sex hormones on renal tubular handling of uric acid [13,28,29]. Evidence for a sex hormone effect is provided by reports showing that the average uric acid level in men is approximately 1 mg/dL higher than that in premenopausal women [30,31,32], and that the prevalence of hyperuricemia is higher in men than in women [33,34]. Since an increase in follicle stimulating hormone (FSH) is a landmark feature of perimenopause followed by a decrease in estradiol [35,36,37], the increased prevalence of hyperuricemia after menopause indicates that the female sex hormone may have protective effects against hyperuricemia. FSH increases 6.1 years before the final menstrual period (FMP), with an acceleration of 2.05 years before the FMP. Obesity attenuates the FSH rise and delays the initial increase [38]. Many studies have shown that the rapid decline in estradiol occurs late in the menopausal transition, particularly in the last 1–2 years [39,40]. These findings support our study results.

The Bio Cycle Study has shown an inverse relationship of progesterone with uric acid level [13], whereas other study found that estradiol treatment did not have any effect on uric acid [41]. The mechanisms underlying the association with female sex hormones are not fully understood. Estradiol is reported to play a more important role than progesterone in the gene expression of uric acid transporters (Urat1, Glut9, and Abcg2) in the mouse kidney [42]. The imbalance of estrogen and progesterone may explain the change in uric acid during the transition period. An age-related increase in serum uric acid level might be a minor factor contributing to the change in estradiol, since the increase in uric acid level among women aged 50 years or older is not observed when adjusting for menopause [43,44].

In the present study, the association between menopausal stage and hyperuricemia was significantly stronger in the obese (BMI ≥25 kg/m^2^) than in non-obese women (BMI <25 kg/m^2^). The interaction between obesity and menopausal stage in affecting hyperuricemia can be explained by the difference in estradiol level; compared with non-obese women, obese and overweight women in premenopausal status have significantly lower estradiol levels, whereas obese women have the higher estradiol level in post-menopause [45]. A steep decrease in estradiol might be expected in the lean body group. The association between menopausal stage and hyperuricemia was stronger in women with a low hsCRP (<1.0 mg/L). This result might be also related to BMI since high hsCRP is reported as an indicator for obesity-related risks [46].

The present study has several limitations. First, the cross-sectional design was used in our study, limiting our ability to establish temporal relationship between menopausal transition and hyperuricemia and its causality. Further longitudinal cohort studies are needed to evaluate the changes in hyperuricemia during the menopausal transition. Second, there might have been misclassification of menopausal stages, which were determined using self-administered questionnaire. Measurement errors of these variables might introduce some degree of residual confounding. Third, we did not measure sex hormone levels in participants of the study. Thus, sex hormones including FSH and/or E2 were not available for the analysis. Further studies including sex hormones are required to elucidate the mechanism underlying the association between menopausal stages and hyperuricemia. Finally, our findings might not be generalized to other populations with other race/ethnicity groups because our study was conducted on middle-aged Korean women who underwent the medical check-up program. However, this study is the first study to demonstrate the increased prevalence of hyperuricemia from late menopause transition using the STRAW+10 classification in a large sample of middle-aged women who experienced a natural menopausal process [20,47].

## 5. Conclusions

Menopausal stages were progressively associated with an increased prevalence of hyperuricemia, especially from the late transition stage. This association was independent of age and other confounders, indicating menopausal stage per se as an independent risk factor for hyperuricemia. The findings of this study merit further investigation with a longitudinal approach to prevent hyperuricemia and its related consequences during menopausal transitions prior to menopause in middle-aged women.

## Figures and Tables

**Table 1:** Characteristics of study participants across menopausal stages.

Characteristics	Overall	Menopausal Stages				*p* for Trend
		Pre-Menopause	Early Transition	Late Transition	Post-Menopause	
**Number of Participants**	58,870	38,356	5637	2614	12,263	
**Uric Acid (mg/dL) ^a^**	4.2 (0.9)	4.2 (0.9)	4.2 (0.9)	4.4 (1.0)	4.5 (1.0)	<0.001
**Age (years) ^a^**	46.9 (7.3)	43.9 (3.6)	43.5 (3.1)	46.3 (4.0)	58.0 (7.0)	<0.001
**Early Menarche (%) ^b^**	2.6	3.0	3.5	2.8	0.7	<0.001
**Parity (%) ^c^**	14.9	10.6	9.1	10.1	32.6	<0.001
**Current Smoker (%)**	2.3	2.3	2.5	2.4	2.4	0.387
**Alcohol Intake (%) ^d^**	11.3	11.8	12.0	9.6	9.2	<0.001
**HEPA (%)**	15.5	14.9	12.3	12.1	19.6	<0.001
**Education Level (%) ^e^**	68.5	76.0	80.9	74.8	36.5	<0.001
**Diabetes (%)**	3.9	2.2	1.7	3.3	10.3	<0.001
**Hypertension (%)**	9.8	5.2	4.8	8.5	26.7	<0.001
**Medication for Dyslipidemia (%)**	4.5	1.5	1.4	2.0	15.7	<0.001
**Body Mass Index (kg/m^2^) ^a^**	22.5 (3.1)	22.2 (3.0)	22.1 (3.0)	23.0 (3.6)	23.5 (3.1)	<0.001
**Systolic Blood Pressure (mmHg) ^a^**	105.7 (12.7)	103.7 (11.7)	104.2 (11.4)	106.9 (13.4)	112.1 (14.2)	<0.001
**Diastolic Blood Pressure (mmHg) ^a^**	67.4 (9.2)	66.6 (9.0)	66.6 (8.8)	68.4 (9.7)	70.1 (9.2)	<0.001
**Glucose (mg/dL) ^a^**	94.0 (13.9)	92.8 (12.3)	92.6 (11.5)	94.1 (14.9)	98.7 (17.7)	<0.001
**eGFR (mL/min/1.73m^2^) ^a^**	101.9 (11.3)	104.2 (10.2)	105.0 (10.1)	102.2 (10.5)	93.0 (10.8)	<0.001
**Total Cholesterol (mg/dL) ^a^**	195.0 (33.8)	191.1 (31.5)	191.3 (31.1)	201.4 (34.8)	207.7 (37.8)	<0.001
**LDL-Cholesterol (mg/dL) ^a^**	120.3 (31.5)	116.2 (29.1)	116.5 (28.8)	126.0 (32.4)	133.6 (35.7)	<0.001
**HDL-Cholesterol (mg/dL) ^a^**	65.3 (15.8)	66.0 (15.6)	66.6 (15.9)	65.2 (15.9)	62.3 (16.0)	<0.001
**Triglycerides (mg/dL) ^f^**	79 (60–109)	76 (58–102)	76 (58–104)	84 (62–118)	92 (67–129)	<0.001
**ALT (u/L) ^f^**	14 (11–19)	13 (11–17)	13 (11–17)	15 (12–21)	18 (14–25)	<0.001
**GGT (u/L) ^f^**	14 (11–20)	13 (11–18)	13 (11–18)	15 (11–22)	17 (13–25)	<0.001
**hsCRP (mg/L) ^f^**	0.3 (0.2–0.7)	0.3 (0.2–0.6)	0.3 (0.2–0.6)	0.4 (0.2–0.9)	0.4 (0.3–0.9)	<0.001
**HOMA-IR ^f^**	1.17 (0.79–1.74)	1.16 (0.79–1.70)	1.14 (0.76–1.69)	1.16 (0.77–1.78)	1.26 (0.81–1.93)	<0.001
**Total Energy Intake (kcal/day) ^f,g^**	1210.5 (875.6–1572.5)	1194.9 (862.6–1557.4)	1083.6 (769.8–1460.0)	1052.5 (754.7–1377.3)	1301.4 (964.4–1642.8)	<0.001

Data are ^a^ the mean (standard deviation); ^b^ <12 years; ^c^ ≥3 times; ^d^ ethanol ≥10 g/day; ^e^ ≥college graduate; ^f^ median (interquartile range). Logistic or linear regression analyses were used as appropriate depending on the type of variables. The number of categories was treated as a continuous variable in regression models in order to test for linear trends; ^g^ among 53,024 participants with plausible estimated energy intake levels (within three standard deviations from the log-transformed mean energy intake). Abbreviations: ALT, alanine transaminase; eGFR, estimated glomerular filtration rate; GGT, gamma-glutamyl transferase; HDL, high-density lipoprotein; HEPA, being health-enhancing physical active; hsCRP, high-sensitivity C-reactive protein; HOMA-IR, homeostasis model assessment of insulin resistance; LDL, low-density lipoprotein.

**Table 2:** Odds ratios (95% confidence intervals) of hyperuricemia (defined as uric acid >6 mg/dL) by menopausal stage.

	Menopausal Stages				*p* for Trend
	Pre-Menopause	Early Transition	Late Transition	Post-Menopause	
**No.**	38,356	5,637	2,614	12,263	
**Cases of Hyperuricemia (%)**	1020 (2.7)	174 (3.1)	140 (5.4)	821 (6.7)	
**Age-Adjusted OR**	1.0	1.18 (1.00–1.39)	1.98 (1.65–2.37)	1.99 (1.71–2.32)	<0.001
**Multivariable-Adjusted OR ^a^**					
**Model 1**	1.0	1.18 (1.00–1.40)	1.67 (1.38–2.03)	1.96 (1.66–2.31)	<0.001
**Model 2**	1.0	1.19 (0.80–1.77)	2.13 (1.35–3.36)	1.65 (1.33–2.04)	<0.001

^a^ Logistic regression model was used. The number of categories was treated as a continuous variable in regression models in order to test for linear trends. Model 1 was adjusted for age, center, year of examination, educational level, smoking status, alcohol consumption, physical activity level, total energy intake, body mass index, parity, and menarcheal age; Model 2: model 1 plus adjustment for medication for hypertension, glucose, systolic blood pressure, triglycerides, high-density lipoprotein cholesterol, low-density lipoprotein cholesterol, homeostasis model assessment of insulin resistance, estimated glomerular filtration rate, and high-sensitivity C-reactive protein. OR, odds ratio.

**Table 3:** Age-matched conditional logistic analysis of hyperuricemia by menopausal stage (*n* = 3356).

	Menopausal Stages				*p* for Trend
	Pre-Menopause	Early Transition	Late Transition	Post-Menopause	
**No.**	2585	78	59	1130	
**Multivariable-adjusted OR ^a^**	1.0	1.36 (0.76–2.44)	2.27 (1.08–4.76)	2.14 (1.55–2.95)	<0.001

^a^ Conditional logistic regression model was used. The number of categories was treated as a continuous variable in regression models in order to test for linear trends. Multivariable model was adjusted for center, year of examination, educational level, physical activity level, smoking status, alcohol consumption, total energy intake, body mass index, parity, and menarcheal age. OR, odds ratio.

**Table 4:** Odds ratios ^a^ (95% Confidence intervals) of hyperuricemia by menopausal stage in clinically relevant subgroups

Subgroup	Menopausal Stages				*p* for Trend	*p* for Interaction
	Pre-Menopause	Early Transition	Late Transition	Post-Menopause		
**Body mass index**						<0.001
**<25 kg/m^2^ (*n* = 48,080)**	1.0	1.05 (0.84–1.33)	1.87 (1.43–2.44)	2.34 (1.95–2.82)	<0.001	
**≥25 kg/m^2^ (*n* = 10,722)**	1.0	1.35 (1.05–1.74)	1.67 (1.28–2.19)	1.47 (1.20–1.79)	<0.001	
**Ever Smoking**						0.72
**No (*n* = 52,068)**	1.0	1.20 (1.00–1.43)	1.68 (1.37–2.05)	1.91 (1.59–2.28)	<0.001	
**Yes (*n* = 2305)**	1.0	0.79 (0.36–1.74)	1.49 (0.63–3.53)	2.05 (1.24–3.40)	0.064	
**Alcohol Intake**						0.69
**<10 g/day (*n* = 47,581)**	1.0	1.22 (1.01–1.47)	1.59 (1.28–1.96)	1.91 (1.58–2.30)	<0.001	
**≥10 g/day (*n* = 6044)**	1.0	1.07 (0.69–1.65)	1.80 (1.07–3.02)	1.62 (1.17–2.25)	0.003	
**Physical Activity**						0.06
**Inactive (*n* = 32,464)**	1.0	1.28 (0.84–1.94)	2.68 (1.72–4.18)	2.13 (1.77–2.56)	<0.001	
**Minimally Active (*n* = 16,876)**	1.0	1.02 (0.54–1.92)	1.49 (0.68–3.26)	1.67 (1.34–2.08)	<0.001	
**HEPA (*n* = 9053)**	1.0	1.17 (0.51–2.72)	3.86 (1.82–8.15)	1.49 (1.15–1.94)	0.001	
**HOMA-IR**						0.35
**<2.5 (*n* = 51,645)**	1.0	1.12 (0.92–1.37)	1.79 (1.43–2.25)	1.91 (1.60–2.28)	<0.001	
**≥2.5 (*n* = 5909)**	1.0	1.42 (1.02–1.98)	1.39 (0.96–2.01)	1.98 (1.55–2.53)	<0.001	
**hsCRP**						0.01
**<1.0 mg/L (*n* = 34,596)**	1.0	1.08 (0.83–0.40)	1.79 (1.32–2.43)	2.12 (1.68–2.68)	<0.001	
**≥1.0 mg/L (*n* = 6859)**	1.0	1.43 (1.06–1.93)	1.26 (0.88–1.80)	1.52 (1.16–1.99)	0.012	

^a^ Logistic regression model was used. The number of categories was treated as a continuous variable in regression models in order to test for linear trends. Likelihood ratio tests were used to test interactions by subgroup comparing models with and without multiplicative interaction terms. Multivariable model was adjusted for age, body mass index, center, year of examination, educational level, smoking status, alcohol consumption, physical activity, total energy intake, parity, and menarcheal age. HEPA, being health-enhancing physical active; HOMA-IR, homeostasis model assessment of insulin resistance; hsCRP, high-sensitivity C-reactive protein.
